# Environmental Contaminants in Hospital Settings and Progress in Disinfecting Techniques

**DOI:** 10.1155/2013/429780

**Published:** 2013-10-30

**Authors:** Gabriele Messina, Emma Ceriale, Daniele Lenzi, Sandra Burgassi, Elena Azzolini, Pietro Manzi

**Affiliations:** ^1^Laboratory of Environmental Hygiene, University of Siena, Italy; ^2^Post Graduate School in Public Health, University of Siena, Italy; ^3^Teaching Hospital “Le Scotte,” Hospital Direction, Siena, Italy

## Abstract

Medical devices, such as stethoscopes, and other objects found in hospital, such as computer keyboards and telephone handsets, may be reservoirs of bacteria for healthcare-associated infections. In this cross-over study involving an Italian teaching hospital we evaluated microbial contamination (total bacterial count (TBC) at 36°C/22°C, *Staphylococcus* spp., moulds, *Enterococcus* spp., *Pseudomonas* spp., *E. coli*, total coliform bacteria, *Acinetobacter* spp., and *Clostridium difficile*) of these devices before and after cleaning and differences in contamination between hospital units and between stethoscopes and keyboards plus handsets. We analysed 37 telephone handsets, 27 computer keyboards, and 35 stethoscopes, comparing their contamination in four hospital units. Wilcoxon signed-rank and Mann-Whitney tests were used. Before cleaning, many samples were positive for *Staphylococcus* spp. and coliforms. After cleaning, CFUs decreased to zero in most comparisons. The first aid unit had the highest and intensive care the lowest contamination (*P* < 0.01). Keyboards and handsets had higher TBC at 22°C (*P* = 0.046) and mould contamination (*P* = 0.002) than stethoscopes. Healthcare professionals should disinfect stethoscopes and other possible sources of bacterial healthcare-associated infections. The cleaning technique used was effective in reducing bacterial contamination. Units with high patient turnover, such as first aid, should practise stricter hygiene.

## 1. Introduction

The Centre for Disease Control (CDC) defines a health-care-associate infections (HAIs) as a “*localized or systemic condition resulting from an adverse reaction to the presence of an infectious agent(s) or its toxin(s). There must be no evidence that the infection was present or incubating at the time of admission to the acute care setting.*” HAIs may be caused by infectious agents from endogenous (body sites) or exogenous sources (patient care personnel, visitors, patient care equipment, medical devices, or the health care environment) [1]. Every year, millions of people across the world suffer from HAIs. HAIs are a wide-ranging concern in the medical field, not only because of morbidity and the possibly of lethal consequences for patients, but also because of extended hospital stays and associated high costs [2–5]. In Europe HAIs cause 16 million extra days of hospital stay and 37000 attributable deaths; they determine approximately costs associated of € 7 billion annually. In the USA around 99000 deaths were attributed to HAIs in 2002 and associated costs were approximately US$ 6.5 billion in 2004 [5, 6].

A Europe-wide point prevalence survey estimated that at least 2.6 million cases of HAI occur annually in long-term care facilities, in addition to ECDC's earlier estimated at 4.1 million patients acquiring HAIs in acute-care hospitals [7].

These infections often have little or nothing to do with the primary reason for the hospital visit but are a result of poor or inadequate hygiene in the healthcare setting [8]. Healthcare equipment is frequently shared between hospital staff, who may have different hygiene practices. Medical devices (stethoscopes, otoscopes, and thermometers) and various objects in hospital environments, such as telephones and computers, have been associated with transmission of HAIs [9–18]. Stethoscopes/phonendoscopes (stethoscopes) are medical devices frequently used in direct contact with patients' skin and can therefore be a vector of infections. These occurrences are known to the scientific community and testified by numerous international studies [9, 11, 16–18]. Physicians should disinfect stethoscopes between one patient and another, though unfortunately this good practice is not always implemented. 

Computers and telephones are now tools of medical practice and are found in all healthcare settings. Their disinfection is often neglected more than that of medical devices. Several studies demonstrate major contamination of these objects and a possible role in the transmission of infection [13, 15]. Current scientific knowledge suggests that the disinfection of environmental surfaces in modern hospitals is indispensable. Furthermore other simple measures, such as hand hygiene of medical staff, remain of a great impact to avoid HAIs [19]. Various studies have investigated singly the role of stethoscopes, computer keyboards, and telephone handsets in HAIs [9–13, 15, 16]. To our knowledge there is lack of research on the evaluation of all these devices together, in different units of the same hospital, where there should be a specific organizational model and risk factors [8] with regard to HAIs. The aims of the present study were to evaluate (i) the contamination of stethoscopes, computer keyboards, and telephone handsets in an Italian teaching hospital before and after use of a disinfecting technique (DT); (ii) differences in contamination in four hospital units; (iii) differences in contamination of medical devices used in clinical practice (stethoscopes) and tools used in medical practice (computer keyboards and telephone handsets).

## 2. Materials and Methods

### 2.1. Setting

We conducted a cross-over study in an Italian teaching hospital with 750 beds in Siena. A variety of hospital environments [8] were chosen to consider different characteristics: “emergency unit” and “first aid” which have a high turnover of patients and disinfection of the environment cannot always be pursued effectively, “intensive care” which observe aseptic conditions and most patients are at high risk of developing infections, and “cardiology/hemodynamic” which are medical units and have rooms with more beds than the intensive care unit and are frequented by many visitors. 

Before the study began, meetings were held between the hospital management and the principal researcher. This is was necessary to explain the project, establish the necessary contacts and avoid any bias in conducting the study. It was considered important to avoid bias caused by doctors/nurses knowing when the investigation would be run, as this might prompt changes in hygiene. It was also decided that stethoscope sampling would be on the same day in each unit, to prevent news of the study circulating and modifying hygienic practices.

### 2.2. Disinfecting Technique

We conducted a surface challenge test to determine the capacity of the disinfectant in killing bacteria and mould [20]. For the cleaning and disinfection of these objects we used a putty compound having a malleable elastic consistency that adheres, removing dirt and disinfecting at the same time. These two characteristics distinguish this disinfecting technique from traditional methods of cleaning and disinfection, aspects normally achieved using two separate operations. The main sanitizing principle of the putty is ethanol (29%) and other components are purified water (51%), guar (6%), glycerine (7%), and minor quantities of other substances, such as boric acid, colorants, and odorants. These features make the putty particularly useful for cleaning and disinfecting surfaces with indentations and protrusions, such as keyboards and handsets. The technique disinfects, as demonstrated by studies conducted according to the indications of the U.S. Pharmacopeial Convention (USP), Chapter 〈1072〉 “*Disinfectants and antiseptic*” and according to CONFARMA protocol number 229100911 A-B which is based on (i) guidelines of the Germany Society for Hygiene and Microbiology 1991, (ii) European standards EN 1040 “*Chemical disinfectants and antiseptics—Basic bactericidal activity—Test method and requirements* (phase 1),” and (iii) EN 13697 “*Chemical disinfectants and antiseptics—Quantitative non-porous surface test for evaluation of bacteria and/or fungicidal activity of chemical disinfectants used in food, industrial, domestic and institutional areas—Test method and requirements without mechanical action* (phase 2/step 2)” [21].

### 2.3. Data Collection

It was decided to study almost all the stethoscopes, computer keyboards, and telephone handsets found in the four units. We analysed 99 objects: 37 telephone handsets, 27 computer keyboards, and 35 stethoscopes (including shared and nonshared ones). 

The experimental protocol required a first sample (swab) H(0) from one-half of each stethoscope membrane, keyboard, and telephone handset, before cleaning with the putty, and a second sample H(1) from the other half of the same objects after cleaning. The swab H(0) was necessary to evaluate the initial contamination level [20]. Samples were obtained by swabbing the surfaces with sterile cotton pads for approximately 5 seconds per stethoscope, 20–30 per keyboard, and 15–20 per telephone. These swabbing times were established according to the different size of the objects. Cleaning half of the stethoscope diaphragm, computer keyboard, and telephone handset with the product took about 20–25 seconds, 2–4 minutes and 20–30 seconds, respectively, depending on dirtiness.

Taking samples, at H(0) and H(1), from both halves of the stethoscopes, keyboards, and telephone handsets was important to avoid the possibility that the first swabs removed bacteria physically, reducing the amount of bacteria collected by the second swab from the same surface and preventing true assessment of product efficacy in reducing bacterial/mould contamination. Because the two halves of the keyboard are different and some keys are used more than others (e.g., “enter”) we decided to alternate the side to which the product was applied. 

All doctors/nurses encountered during the visit to the units were informed by the principal researcher/hospital management doctor of the study and were asked if there was any problem about carrying out the study; there were no objections. A new pack of product was used for every object. The following information was also recorded at the time of sampling: hospital identification (ID), department ID, and doctor/nurse ID. Records were indexed with a unique ID for each sample. The same ID was assigned to the pack of cleaning putty. All the information was recorded and stored in a database for future analysis.

### 2.4. Laboratory Analysis

Analysis was carried out in the Hygiene and Environmental Laboratory of the University of Siena, where the swabs were placed in 1 mL phosphate buffered saline, shaken in a vortex mixer and the liquid sown (0.1 mL/plate) in Petri dishes containing plate count agar (PCA) for total microbial load incubating at 36°C for mesophilic germs (human contamination) and at 22°C for psychrophilic microorganisms (environmental contamination). Index microorganisms were cultured in mannitol salt agar for *Staphylococcus* spp. (hand-transmitted pathogens),* Pseudomonas* cetrimide for *Pseudomonas* spp. faecal contamination), Slanetz & Bartley medium for *Enterococcus* spp. (faecal contamination), Brilliance *E. coli/coliform* spp. chromogenic medium for *Escherichia coli* and coliform bacteria (faecal contamination), *Acinetobacter* base for *Acinetobacter* spp. (an emerging alert for HAIs), and Brilliance methicillin-resistant *Staphylococcus aureus* (MRSA) MRSA2 medium for methicillin-resistant *Staphylococcus aureus* incubating at 36°C (*Staphylococcus aureus *resistant to methicillin antibiotics). *Clostridium difficile* agar base was supplemented with *Clostridium difficile* selective supplement and 7% defibrinated horse blood for *Clostridium difficile* spp. (as an indicator of poor disinfection) with incubation for 48 hours at 36°C in an anaerobiosis jar. Anaerobiosis was obtained using a gas generating kit. 

All the sowings were made by the same technician of the Department of Physiopathology, Experimental Medicine and Public Health involved in the study. The Petri dishes were read by the principal researcher and the technician. The results were expressed as colony-forming units per swab (CFU/0.1 mL). The plates were read 24 and 48 hours after sowing. We opted for a double count: at 24 hours to prevent vigorous bacterial growth from rendering some colonies uncountable at 48 hours and at 48 hours to avoid missing bacterial species/colonies with slower growth. All bacteria/mould counts were added to the previous database for further use.

### 2.5. Statistical Analysis

Database cleaning was performed before data analysis. Descriptive analysis (mean, standard deviation, median, interquartile range, minimum, and maximum) of the data for all types of microbes/moulds was performed for H(0) and H(1). For each bacterium, we counted the number of positive samples for H(0) and H(1) and calculated their percentages. We also calculated the total quantitative CFU count of the 99 objects for H(0) and H(1) and the percentage reduction after use of the experimental disinfecting technique. The matched approach used (two halves of each object, one before and one after cleaning) aimed at better control of confounders, minimizing their effects and increasing the reliability of the results. When CFUs of H(1) were not zero, statistical tests were carried to highlight differences between before and after cleaning. Descriptive analysis for H(0) was also conducted at ward level to determine contamination load. To reveal differences in bacterial contamination before and after use of the product the Wilcoxon signed-rank test was used, while the Mann-Whitney test was used to detect differences (i) between units, (ii) between telephone handsets plus computer keyboards versus stethoscopes, and (iii) between personal and shared stethoscopes [22].

Significance was set at *P* < 0.05. Stata SE, version 12.1 software (StataCorp, College Station, TX, USA), was used for the analysis.

## 3. Results

Descriptive statistics are shown in Table 1. Mean, median, interquartile range, and standard deviation were obtained considering only positive samples. The percentage of positive H(0) samples was generally higher on computer keyboards, followed by telephone handsets and stethoscopes. The only exception was MRSA, where the latter had a higher percentage of positive samples: 28.6% compared to 16.2% for telephone handsets and 22.2% for computer keyboards.

No H(0) or H(1) samples contained *Pseudomonas* or *Clostridium difficile* (only investigated on stethoscopes). CFUs decreased to zero in most comparisons.

For stethoscopes, significant CFU differences were detected in PCA 36, PCA22, *Staphylococcus* spp. (*P* < 0.0001), *E. coli* (*P* = 0.025), *Coliforms* (*P* = 0.0001), MRSA (*P* = 0.002), and *Enterococcus* spp. (*P* = 0.046). For the latter, only four samples were positive before cleaning. No CFU differences emerged for moulds (*P* < 0.563); only 2 stethoscopes were contaminated with moulds before cleaning and one after cleaning. 

For computer keyboards we found CFU differences before and after cleaning for PCA 36, PCA22, *Coliforms*, *Staphylococcus* spp., moulds (*P* < 0.0001), *E. coli* (*P* = 0.001), *Enterococcus *spp. (*P* = 0.045) and MRSA (*P* = 0.014). One keyboard H(0) sample was contaminated with *Acinetobacter* spp.

No significant differences were identified in the distribution of telephone handsets, computer keyboards and stethoscopes in the 4 wards (*P* = 0.325, Fisher's exact *X* ^2^).

For telephone handsets, significant differences in CFUs were highlighted for PCA36, PCA22, *Coliforms*, *Staphylococcus* spp. (*P* < 0.0001), and MRSA (*P* = 0.008). Six telephone handsets were contaminated with moulds before cleaning and one still after cleaning. 

Our results showed that the highest CFU counts came from the “first aid” unit and the lowest ones from the “intensive care” unit. Differences between these two units were significant for all bacterial species (*P* < 0.01).  Table 2 shows H(0) bacteria/mould contamination counts (mean, standard deviation, median, and interquartile range) for the four units and comparisons between them by the Mann-Whitney test.

Comparisons of CFUs between computer keyboards plus telephone handsets versus stethoscopes showed significant differences: higher contamination was always found on computer keyboards and telephone handsets than on stethoscopes for moulds (*P* = 0.002). For PCA 36°C and PCA 22°C we obtained borderline results: *P* = 0.064 and *P* = 0.046, respectively.

In some cases, comparisons between shared stethoscopes with nonshared ones also showed significant differences in CFUs. We found greater contamination on nurses' and physicians' stethoscopes by PCA 36°C (*P* = 0.015) and *Staphylococcus* spp. (*P* = 0.016); for PCA 22°C and MRSA the differences were borderline significant (*P* = 0.063 and *P* = 0.047, resp.).

## 4. Discussion

Computer keyboards and telephone handsets are commonly found in hospital environments. Several studies have reported contamination of these objects and their possible role in the transmission of HAIs [9–18]. Our study, unlike others, considered all these items together in a hospital setting, as well as comparing units with different organization, patient turnover, medical personnel, and visitors. 

In line with our results, other studies also isolated gram-positive cocci [16, 17, 23–25], especially *Staphylococcus* spp. In our study, 21/35 stethoscopes, 25/27, computer keyboards, and 31/37 telephone handsets were positive for these bacteria. Among H(0) samples, 10/35, 6/27 and 7/37 were positive for MRSA, respectively. We also isolated several bacterial species that are often the cause of HAIs, such as *Coliforms* and *Enterococcus* spp. *Acinetobacter* spp. was also isolated from one computer keyboard. 

After cleaning with the putty containing about 30% ethyl alcohol, CFUs decreased to zero in most cases and by percentages higher than 96% for PCA 36°C (for all objects); moulds (for computer keyboards); PCA 22°C, *Coliforms and Staphylococcus* spp. (for telephone handsets and computer keyboards). Moulds decreased from two to one positive sample in stethoscopes and from 14 to 3 in telephone handsets (−78, 6%). 

Other studies investigating the effectiveness of different sanitizing techniques in reducing microbial contamination of stethoscopes [17, 26–29] looked at products such as ethyl alcohol and ethanol-based cleaners, isopropyl and isopropyl-based compounds, and antiseptic soaps, all of which showed the same or less effectiveness than that of the present product [17, 26–28]. On the contrary, few studies have been conducted on cleaning techniques for telephone handsets and computer keyboards [15], where disinfection is more complex than that for stethoscopes. In fact, liquid compounds may cause aesthetic and functional damage, especially to objects with electronic circuits. The putty product penetrated between and under the keys of keyboards and into the holes of telephone handsets, without wetting the surfaces. 

In H(0) samples our results showed that although the percentage of stethoscopes contaminated by bacteria/moulds was lower than of that telephone handsets and computer keyboards, mean and median CFUs were higher in stethoscope samples (Table 1). This finding is also significant because stethoscopes are much easier to clean. This apparent contradiction (lower contamination percentages but higher numbers of CFUs) could mean that while healthcare personnel are certainly aware of the need to clean/disinfect stethoscopes, the practice is sometimes neglected. Neglecting cleaning and disinfection of telephone handsets and especially computer keyboards can have negative consequences. In fact, although our results confirmed a lower number of CFUs on these objects than on stethoscopes, the percentages of contaminated objects, H(0) samples, were generally much higher, indicating an aptitude as reservoir of infection. The only *Acinetobacter* spp. found, albeit a single CFU, was on a computer keyboard and its presence could mean that (i) healthcare staff pay more attention to disinfecting “real” medical devices than other objects; (ii) it is easier to disinfect smooth surfaces, such as stethoscopes, than surfaces with indentations and protrusions, like keyboards; (iii) healthcare staff underestimate the possibility that contamination can occur from objects which are not strictly considered medical devices. It has been demonstrated that MRSA can survive up to seven months on inanimate surfaces, vancomycin-resistant germs up to four months, *Acinetobacter* spp. up to five months, *Klebsiella* spp. up to thirty months, and spores of *Clostridium difficile* up to five months [8]. 

Neglecting the cleaning/disinfection of computer keyboards and the long survival of some bacteria are critical in the role of HAIs. In fact, although healthcare professionals may clean and disinfect their “personal” medical devices, they may still be contaminated by hospital computer keyboards and telephone handsets and transmit infections. Both these devices are used with the hands, which are the top causes of transmission of bacteria [8, 30, 31]. Frequency of disinfection and compliance of hospital personnel could explain the differences we found between the units. For example, “intensive care” was understandably the least contaminated (Table 2), due to strict observance of protocols for aseptic conditions, rooms with few beds, and exclusion of visitors. Moreover, the staff has instructions to clean stethoscopes after each use. Keyboards and telephone handsets are used by staff particularly conscientious about disinfection practices. The “first aid” unit understandably had the highest level of contamination due to fast patient turnover and the greater number of doctors, nurses, and visitors [8]. Here, the staff seemed less sensitive to hygiene, prioritizing “strictly medical activities” and emergency procedures. 

Another study [32], involving an intensive care unit, found low bacterial contamination of stethoscopes. These researchers investigated stethoscopes' pathogenic bacterial and skin flora contamination on diaphragms of 46 stethoscopes (22 personal and 24 bedside ones). They found that bedside stethoscopes had pathogenic bacteria and skin flora in 2 and 14 samples, respectively, and the personal ones had the same organisms in 3 and 18 samples, respectively.

We also compared microbial contamination of stethoscopes of physicians/nurses and shared stethoscopes and found some differences. We recorded greater contamination on nonshared stethoscopes by PCA 36 (*P* = 0.015), PCA 22 (*P* = 0.063), *Staphylococcus* spp. (*P* = 0.016), and MRSA (*P* = 0.047). Healthcare professionals presumably use their own stethoscopes more often than shared ones and do not clean them very often, so that they are more likely to be contaminated. Other reasons for greater contamination of personal stethoscopes could be that shared stethoscopes are subject to established hygiene practices and procedures while personal ones are less likely to be cleaned and disinfected by the owner.

This study involved hospital units with different features and it focused on devices with different structures, surfaces, and functions. These aspects could distort certain comparisons; in particular: (i) the distribution of computer keyboards, telephone handsets, and stethoscopes was not the same in the four units studied, which could influence the number of contaminated samples. However, statistical analysis indicated that these differences in distribution of devices seemed not significant (*P* = 0.325, Fisher's exact *X* ^2^) and this problem should be avoided; (ii) the swab taken from the half-surface of a computer keyboard is larger than one from half a stethoscope membrane. We therefore expected a greater number of CFUs from keyboards than from stethoscopes but we actually found the opposite. There were, however, more positive samples from computers and telephones than from stethoscopes, and this was expected due the larger surface areas swabbed. In addition while stethoscopes were presumably disinfected more often and more easily than the other devices, their function determined a greater likelihood of contamination. 

## 5. Conclusions

In conclusion, our results highlight the importance of proper cleaning/disinfection of medical devices, as well as typical office equipment (computer keyboards and telephone handsets), used by medical staff. All can play a role in bacterial contamination and transmission as a possible cause of HAIs. The disinfecting technique used was effective in reducing/eliminating bacterial load, even on computer keyboards, which were the most complicated of the objects considered to clean/disinfect. The emergence of resistant bacteria, hospitalization of older and critical patients, high turnover of healthcare staff making process standardization difficult, lack of process organization between units and nonobservation of protocols [8] are all areas in need of improvement to prevent HAIs. Environmental disinfection has a central role in the primary prevention of HAIs.

## Figures and Tables

**Table 1:** Descriptive statistics of stethoscopes, computer keyboards, and telephone handsets at H(0) and H(1): number and percentage of positive samples, overall CFUs counts and percentage reduction in CFUs from H(0) to H(1), means, standard deviations, medians, interquartile ranges (IQR), minima, and maxima.

Object	Culture medium*	Time	Positive sample (%)	CFU total count^b^	% reduction	Mean^a ^(SD)	Median^a^ (IQR)	Min^a^	Max
Stethoscopes (*n* = 35)	TBC 36	H(0)	20 (57.1)	3368	−99.97	168.4 (304.7)	19 (7.5 to 188)	1	1110
		H(1)	1 (2.9)	1		1 (—)	1 (1)	1	1
	TBC 22	H(0)	22 (62.9)	3678	−100	167.2 (367)	9.5 (5 to 55)	1	1508
		H(1)	0 (0)	0		— (—)	— (—)	—	—
	*E. coli *	H(0)	5 (14.3)	123	−100	24.6 (30)	11 (3 to 37)	1	71
		H(1)	0 (0)	0		— (—)	— (—)	—	—
	*Coliforms *	H(0)	15 (42.9)	1009	−100	67.2 (120)	6 (3 to 62)	1	361
		H(1)	0 (0)	0		— (—)	— (—)	—	—
	*Enterococci *	H(0)	4 (11.4)	14	−100	3.5 (4.4)	1.5 (1 to 6)	1	10
		H(1)	0 (0)	0		— (—)	— (—)	—	—
	*Staphylococci *	H(0)	21 (60)	2605	−100	124.1 (270)	10 (5 to 70)	2	1003
		H(1)	0 (0)	0		— (—)	— (—)	—	—
	MRSA	H(0)	10 (28.6)	130	−100	13 (16)	5 (2 to 21)	1	50
		H(1)	0 (0)	0		— (—)	— (—)	—	—
	Moulds	H(0)	2 (5.7)	2	−50	1 (—)	1 (1)	1	1
		H(1)	1 (2.9)	1		1 (—)	1 (1)	1	1

Telephone handsets (*n* = 37)	TBC 36	H(0)	33 (89.2)	645	−99.22	20 (30)	11 (4 to 25)	1	156
		H(1)	3 (8.12)	5		1.7 (0.6)	2 (1 to 2)	1	2
	TBC 22	H(0)	33 (89.2)	580	−98.79	18 (28.5)	6 (2 to 22)	1	152
		H(1)	4 (10.8)	7		1.8 (1.5)	1 (1 to 2.5)	1	4
	*E. coli *	H(0)	1 (2.7)	1	−100	1 (—)	1 (1)	1	1
		H(1)	0 (0)	0		— (—)	— (—)	—	—
	*Coliforms *	H(0)	22 (59.5)	194	−99.48	8.8 (7.8)	8 (2 to 12)	1	30
		H(1)	1 (2.7)	1		1 (—)	1 (1)	1	1
	*Enterococci *	H(0)	2 (5.4)	2	−100	1 (—)	1 (1)	1	1
		H(1)	0 (0)	0		— (—)	— (—)	—	—
	*Staphylococci *	H(0)	31 (83.8)	427	−99.53	13.8 (22.4)	6 (2 to 14)	1	99
		H(1)	2 (5.4)	2		1 (—)	1 (1)	1	1
	MRSA	H(0)	7 (18.9)	23	−100	3.3 (2.8)	3 (1 to 4)	1	9
		H(1)	0 (0)	0		— (—)	— (—)	—	—
	Moulds	H(0)	6 (16.2)	14	−78.57	2.3 (2.4)	1 (1 to 3)	1	7
		H(1)	1 (2.7)	3		3 (—)	3 (3)	3	3

Computer keyboards (*n* = 27)	TBC 36	H(0)	26 (96.3)	512	−99.41	19.7 (16.6)	14 (8 to 33)	1	66
		H(1)	2 (7.4)	3		1.5 (0.7)	1.5 (1 to 2)	1	2
	TBC 22	H(0)	25 (92.6)	557	−96.77	22.3 (15.2)	21 (9 to 31)	1	57
		H(1)	4 (14.8)	18		4.5 (5.7)	2 (1.5 to 7.5)	1	13
	*E. coli *	H(0)	11 (40.7)	16	−100	1.5 (0.7)	1 (1 to 2)	1	3
		H(1)	0 (0)	0		— (—)	— (—)	—	—
	*Coliforms *	H(0)	21 (77.8)	147	−96.60	7 (6.5)	5 (3 to 9)	1	27
		H(1)	2 (7.4)	5		2.5 (2.1)	2.5 (1 to 4)	1	4
	*Enterococci *	H(0)	4 (14.8)	4	−100	1 (—)	1 (1)	1	1
		H(1)	0 (0)	0		— (—)	— (—)	—	—
	*Staphylococci *	H(0)	25 (92.6)	239	−99.58	9.6 (9)	5 (4 to 12)	1	37
		H(1)	1 (3.7)	1		1 (—)	1 (1)	1	1
	MRSA	H(0)	6 (22.2)	27	−100	4.5 (3)	4.5 (2 to 6)	1	9
		H(1)	0 (0)	0		— (—)	— (—)	—	—
	Moulds	H(0)	20 (74.1)	137	−99.27	6.9 (8)	3.5 (1.5 to 9)	1	29
		H(1)	1 (3.7)	1		1 (1)	1 (1)	1	1

*1 CFU of *Acinetobacter* was found only in one keyboard at H(0); TBC 36 and TBC 22, respectively, are total microbial load incubating at 36°C and 22°C; MRSA: methicillin-resistant *Staphylococcus aureus*; ^a^only positive samples; ^b^summing all CFUs per stethoscopes, computer keyboards, and telephone handsets.

**Table 2:** Number of CFUs in H(0) samples from the four hospital units: median, interquartile range (IQR), mean and standard deviation *P* value comparing data of the four units by the Mann-Whitney test.

Culture medium	(A): cardiology and hemodynamic, 33 objects		(B): first aid, 25 objects		(C): intensive care unit, 29 objects		(D): emergency unit, 12 objects		*P* value comparing data of the four units by the Mann-Whitney test					
	Median (IQR)	Mean (SD)	Median (IQR)	Mean (SD)	Median (IQR)	Mean (SD)	Median (IQR)	Mean (SD)	A versus B	A versus C	A versus D	B versus C	B versus D	C versus D
TBC 36*	9 (4 to 27)	63.0 (157.6)	24 (8 to 39)	41.52 (86.9)	2 (0 to 12)	6.96 (9.8)	7 (2.5 to 10)	100.6 (318.1)	0.136	0.005	0.232	0.0007	0.073	0.224
TBC 22	9 (3 to 26)	63.7 (170.5)	27 (5 to 41)	37.2 (69.8)	2 (0 to 9)	6.14 (11.2)	6 (0.5 to 17)	133.8 (432.9)	0.095	0.002	0.233	0.0001	0.066	0.181
*E. coli *	0 (—)	1.7 (6.6)	0 (0 to 1)	0.4 (0.8)	0 (—)	0.03 (0.2)	0 (—)	6.1 (20.5)	0.048	0.035	0.058	0.011	0.043	0.089
*Coliforms *	1 (0 to 5)	19.9 (68.5)	8 (5 to 14)	11.8 (13.6)	0 (0 to 2)	1.4 (2.8)	1.5 (0 to 9)	29.8 (90.1)	0.008	0.006	0.680	<0.001	0.088	0.128
*Staphylococci *	5 (2 to 14)	44.7 (145.5)	12 (3 to 25)	23.7 (36.6)	2 (0 to 3)	4.8 (13.0)	6.5 (2.5 to 9.5)	88.7 (288.0)	0.01	0.002	0.533	0.006	0,101	0.041
MRSA**	0 (—)	3 (10.0)	0 (0 to 2)	2.0 (4.5)	0 (—)	0.2 (0.7)	0 (0 to 5)	2.3 (3.2)	0.088	0.114	0.093	0.004	0.459	0.003
Moulds	0 (0 to 1)	1.4 (4.1)	0 (0 to 3)	2.4 (4.3)	0 (—)	0.07 (0.3)	0 (0 to 1.5)	3.8 (8.9)	0.224	0.004	0.551	0.001	0.317	0.082

*TBC 36 and TBC 22: total bacterial count incubating at 36°C and 22°C, respectively; **MRSA: methicillin-resistant *Staphylococcus aureus*.
